# Clinical Applications for Cardiovascular Magnetic Resonance Imaging at 3 Tesla

**DOI:** 10.2174/157340309788970351

**Published:** 2009-08

**Authors:** Allison G Hays, Michael Schär, Sebastian Kelle

**Affiliations:** 1 Department of Medicine, Division of Cardiology, Johns Hopkins University, Baltimore, Maryland, USA; 2 Philips Healthcare, Cleveland, Ohio, USA; 3 Department of Radiology, Division of Magnetic Resonance Research, Johns Hopkins University, Baltimore, Maryland, USA; 4 Department of Medicine, Division of Cardiology, Deutsches Herzzentrum Berlin, Germany

**Keywords:** 3 Tesla, cardiac magnetic resonance.

## Abstract

Cardiovascular magnetic resonance (CMR) imaging has evolved rapidly and is now accepted as a powerful diagnostic tool with significant clinical and research applications. Clinical 3 Tesla (3 T) scanners are increasingly available and offer improved diagnostic capabilities compared to 1.5 T scanners for perfusion, viability, and coronary imaging. Although technical challenges remain for cardiac imaging at higher field strengths such as balanced steady state free precession (bSSFP) cine imaging, the majority of cardiac applications are feasible at 3 T with comparable or superior image quality to that of 1.5 T. This review will focus on the benefits and limitations of 3 T CMR for common clinical applications and examine areas in development for potential clinical use.

## INTRODUCTION

Cardiovascular magnetic resonance (CMR) has become a widely adapted imaging modality for the diagnosis of cardiovascular disease, and its clinical indications have expanded greatly in the last decade [1]. Recently, 3 T CMR has become available and has demonstrated advantages over 1.5 Tesla over a broad range of clinical applications, for example perfusion imaging [2-5]; delayed enhancement [6, 7]; myocardial tagging [8, 9]; and coronary magnetic resonance imaging [10]. This review will focus on the recent advances and clinical utility of 3 T CMR and the potential limitations of the technique. 

## CARDIAC FUNCTION

**Fig. (1):**
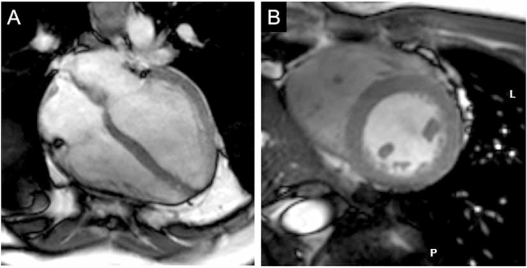
Balanced steady-state free precession (bSSFP) ventricular function images acquired with a 32 channel receiver coil at 3T. **(A)** 4-chamber view and **(B)** basal short-axis view. (in collaboration with Ashraf Hamdan (MD), Berlin).

For cardiac cine imaging, balanced steady-state free precession (bSSFP) techniques are employed at 1.5 T and are considered the gold standard for evaluating cardiac function [11]. The application of accelerated parallel imaging techniques employing multiple receive coils show that bSSFP cine imaging at 3 T is at least comparable to that of 1.5 T [12, 13]. However, at higher magnetic field strengths, there are technical challenges associated with cardiac bSSFP imaging because of increased inhomogeneities of the static magnetic field (B_0_) [14]. High signal-to-noise ratio (SNR) and contrast-to-noise ratio (CNR) between blood and cardiac muscle in bSSFP imaging comes at the cost of so called “dark band” artifacts. These artifacts appear further away from the determined imaging frequency when a shorter imaging repetition time (TR) is employed [15]. They degrade overall image quality [16] and one group observed off-resonance artifacts in 86% of subjects [6]. There are two steps to be taken to minimize dark band artifacts [17]: First, the TR needs to be chosen as short as possible to push the bands away from the heart. Short TRs, however, are challenged by the increased energy deposition at 3 T compared to 1.5 T and the desire for high excitation angles and long readouts to increase contrast and resolution, respectively. Second, B_0_ field homogeneity should be optimized over the heart with localized second order shimming and correct determination of the imaging frequency [17-19]. Although bSSFP cine imaging at 3 T is feasible (Fig. **1**), currently implemented shim routines do not completely shift dark band artifacts from cardiac structures in all cases [13]. 

Spoiled gradient echo (GRE) techniques are applied at 3 T for cine imaging and have shown superior results in comparison to 1.5 T [20, 21]. A major disadvantage of GRE techniques for cine imaging is the relative dependence on inflow for contrast between the myocardium and blood, which may lead to reduced contrast particularly in the long axis views. Therefore, if the blood flow is depressed such as in severe left ventricular (LV) dysfunction, the blood becomes saturated, resulting in lower contrast between the chamber and ventricular wall. In GRE techniques both extravascular and intravascular contrast agents have been employed to improve endocardial border definition for the accurate quantification of wall motion [22, 23]. A practical approach is to perform LV function imaging shortly after contrast administration with gadolinium and before obtaining delayed enhancement images [23].

## TAGGING

Myocardial tagging has been increasingly employed for the accurate, semi-automated analysis of myocardial wall motion and strain measurement [24]. The technique is used to track the deformation of a presaturation line or grid throughout the cardiac cycle. When combined with cine imaging, myocardial tagging provides a quantitative estimation of regional wall motion abnormalities, particularly when combined with strain-encoded techniques or harmonic phase methods [25]. One of the limitations of tagging is that tag lines fade during end-diastole. Several studies have shown that myocardial tagging techniques are improved at 3 T compared to 1.5 T due to higher SNR, CNR and reduced fading of tags in diastole because of the increased T1 at higher field strengths [8, 9, 26]. Newer advances such as the use of real time fast strain-encoded MRI (fast-SENC) has been employed to acquire images in a single heartbeat without the need for breath hold techniques, which is valuable for the study of patients. Fast-SENC was shown to be superior to conventional tagging techniques for the assessment of regional myocardial function at 3 T [27]. In addition, strain-encoding techniques at 3 T may be useful for the evaluation of right-ventricular regional function [28], which has traditionally been challenging. 

## CMR PERFUSION

**Fig. (2):**
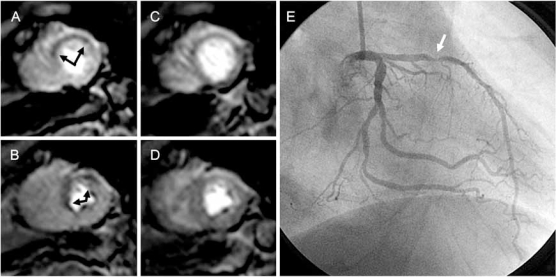
Patient with stress-inducible anterior and anteroseptal perfusion defect (black arrows). Scans show **A**, apical and **B**, equatorial shortaxis views during stress; **C** and **D** show perfusion images at rest. *E,* Coronary angiography shows 90% stenosis (white arrow) of proximal LAD. With permission of [2].

Myocardial perfusion magnetic resonance (MR) imaging has evolved considerably over the past decade and is used to assess the significance of coronary artery stenosis and microvascular dysfunction on the myocardium. Clinical studies performed with 1.5 T scanners have shown that MR perfusion imaging yields superior diagnostic results for the detection of CAD to clinically established nuclear perfusion techniques [29-31]. By capturing the first pass of a contrast agent using an inversion-recovery gradient echo technique or equivalent, high temporal resolution images can be generated that provide a valuable diagnostic tool particularly for the evaluation of intermediate risk patients with chest pain [1]. However, because perfusion imaging requires a fast acquisition time, it is performed with relatively low spatial resolution at 1.5 T which may cause dark rim artifacts that may be mistaken for areas of hypoperfusion. Perfusion imaging benefits from the high SNR achievable at 3 T (Fig. **2**) and may reduce the occurrence of dark ring artifacts. Significant improvements in SNR and overall image quality have been reported for perfusion imaging at 3 T [3, 26, 32]. One study showed that the diagnostic accuracy of 3 T perfusion imaging with adenosine is superior to that of 1.5 T (90% vs. 82%) when identifying patients with significant coronary artery stenoses [33]. A recent report demonstrated that the abundant spatiotemporal correlation enables highly accelerated perfusion MR imaging with high spatial resolution at 3 T and improves SNR and image quality compared with those at 1.5 T. Compared with perfusion MR imaging at lower spatial resolution, image quality was improved and artifacts were reduced [34]. 

## DOBUTAMINE CMR

Dobutamine stress CMR has become a well established modality for the diagnosis of myocardial ischemia. It has improved sensitivity and specificity for the detection of myocardial ischemia when compared with other stress techniques such as dobutamine stress echocardiography and is beneficial in patients with poor acoustic windows for echocardiography [35]. Dobutamine CMR has a powerful prognostic value for patients with suspected or known CAD and it has a high negative predictive value for future cardiovascular events [36]. A recent study examined the feasibility and accuracy of stress imaging at 3 T using high dose dobutamine. In patients with suspected or known coronary artery disease (CAD), resting cine images using a spoiled gradient echo technique were performed immediately after the administration of gadolinium to improve image quality. This study reported a sensitivity and specificity of 80.0% and 85.7%, respectively, for the detection of significant flow-limiting coronary stenosis as defined on cardiac catheterization [37]. The implementation of parallel imaging will likely further enhance the temporal and spatial resolution, as well as accuracy of stress protocols in the future. 

## LATE GADOLINIUM ENHANCEMENT

The measurement of late gadolinium enhancement (LGE) of the myocardium using gadolinium has been broadly accepted in recent years as the imaging method of choice to evaluate myocardial scar [38]. The technique of LGE has been validated as a means to assess the amount of nonviable myocardium as a percentage of the transmural extent in a given segment and is inversely related to the likelihood of functional recovery after revascularization [39]. The degree and extent of myocardial scar as measured by MRI has a strong predictive value for future cardiovascular events [40]. The higher achievable SNR at 3 T may benefit cardiac scar imaging by providing higher contrast between healthy and diseased (non-viable) myocardium. In patients with a history of myocardial infarction, a higher image quality of LGE was demonstrated at 3 T compared to 1.5 T [6]. Another group performed intra-subject comparisons in patients with a history of acute and chronic myocardial infarction using the same contrast-enhanced viability protocol at both 1.5 and 3 T, and found very close agreement for myocardial enhancement [7]. 

**Fig. (3):**
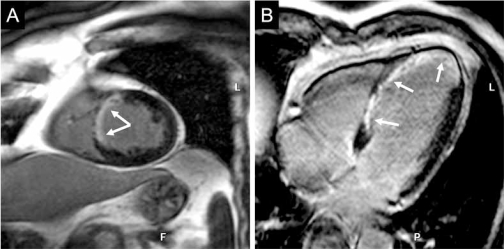
Late gadolinium enhancement images of a patient with chronic myocardial infarction of the septal ventricular wall, including the apex (white arrows). Images in basal short-axis view **(A)** and 4-chamber view **(B)** at 3 T acquired 15 minutes after injection of 0.15 mmol gadolinium/kg body weight.

There are several recent studies that examine potential benefits of 3 T imaging in LGE (Fig. **3**). One advantage is the possibility of reducing the dose of contrast agent at 3 T [41]. In addition, the higher spatial resolution may help to better delineate infarct zones from peri-infarct regions, which may be a focus of ventricular arrythmias and has been reported to be a strong predictor of future cardiovascular events [42]. Recently, newer methods such as stimulated-echo acquisition mode (STEAM) MRI have been implemented at 3 T for black-blood LGE myocardial imaging [43]. This method demonstrates good agreement with standard inversion recovery LGE imaging and allows for improved determination of the blood-infarct border which may enhance the measurement of infarct size. 

## CORONARY MRI

Coronary magnetic resonance angiography (MRA) provides a non-invasive, safe means to evaluate the coronary arteries, and may be improved at 3 T [44]. An initial study of coronary angiography at 3 T in healthy adults reported a higher spatial resolution compared to that achievable at 1.5 T [10]. One group reported an intraindividual comparison of 3D bSSFP coronary MRA using both field strengths and found a 93% increase in CNR at 3 T compared to 1.5 T [45]. Sommer and coworkers directly compared 3 T to 1.5 T coronary MRA to assess the accuracy of diagnosing coronary artery disease compared to the current “gold standard” of coronary angiography [46]. Using navigator-corrected, 3D turbo gradient-echo techniques at both field strengths, they found comparable image quality with a 30% increase in SNR and a 22% increase in CNR at 3 T. Overall, the diagnostic accuracy at both field strengths was equivalent, with sensitivity for the detection of CAD of 82% for both, and a specificity of 89% and 88% for 3 T and 1.5 T, respectively. However, newer techniques that were not employed at the time such as optimized T2 preparation pulses [47], parallel imaging [48], or advanced shimming algorithms [17] will likely contribute to superior results of coronary MRA at higher field strengths. 

The use of CT to perform coronary angiography for the diagnosis of CAD has been progressing rapidly. A recently performed multi-center trial using multi-detector 64-Row CT reported a sensitivity of 85% and a specificity of 90% in detecting coronary stenoses > 50% on x-ray coronary angiography [49]. Heavily calcified vessels still present a hurdle to overcome for CT angiography, as the lumen cannot be visualized in a significant number of these segments. Overall, the diagnostic accuracy for the detection of significant CAD continues to favor the use of CTA over MRA.

A recent meta-analysis of 51 studies that each examined coronary CTA or MRA reported a significantly higher sensitivity and specificity for CTA (85%, 95% respectively) compared with that of coronary MRA (72%, 87% respectively) for the detection of significant coronary stenoses [50]. 

Actually, there are no data available comparing state of the art coronary CT and coronary MRA at 3 T, e.g. favoring the use of a 32-channel receiver coil and a contrast-enhanced whole-heart approach [51].

## CORONARY VESSEL WALL IMAGING

**Fig. (4):**
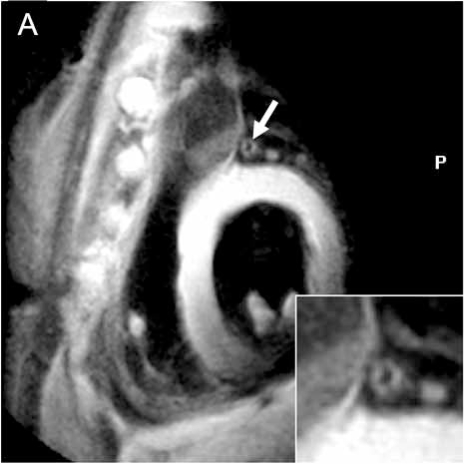
**(A)** Black-blood image at 3 T of the proximal left anterior descending (LAD) (white arrow) in a CAD patient shows thickened coronary vessel wall (zoomed image – white arrow).

Coronary vessel wall imaging using CMR permits the non-invasive quantification for “Glagov-type” outward arterial remodeling and allows for the accurate measurement of vessel wall thickness [52]. Because thickening of the vessel wall precedes luminal narrowing, MRI has the ability to detect early coronary atherosclerosis (Fig. **4**). In a study of patients with CAD, a free-breathing, navigator-gated technique for 3D coronary black blood imaging showed increased coronary vessel wall thickness in patients with mild CAD when compared to a healthy control population [53]. Preliminary studies of coronary vessel wall imaging at 3 T are promising and show the potential to detect preclinical disease and monitor treatment effects over time [52, 54]. 

## SPECTROSCOPY

Cardiac MR spectroscopy (MRS) enables the non-invasive assessment of high-energy phosphate metabolism and even flux through the creatine kinase reaction [55, 56] when applying phosphorus (^31^P) MRS [57-61] or the myocardial triglyceride content with proton (^1^H) MRS [62-64]. Currently, however, cardiac MRS clinical use is limited, in large part to the inherent low concentration of the metabolites being studied which in turn restricts the achievable spatial resolution and often imposes long scan times [65]. Cardiac MRS at high field may profit from both increased SNR and spectral dispersion [66, 67] and will offer a powerful means to non-invasively probe critical metabolic processes in human heart disease.

## SUMMARY

Cardiac MR imaging has evolved rapidly and is now accepted as a powerful diagnostic tool with significant clinical and research applications. Although technical challenges remain for cardiac imaging at higher field strengths such as bSSFP cine imaging, the majority of cardiac applications are feasible at 3 T with comparable or superior image quality to that of 1.5 T. Imaging at 3 T particularly benefits protocols with sub-optimal SNR such as cardiac perfusion imaging, delayed enhancement imaging and myocardial tagging techniques. The integration of parallel imaging, motion compensation, and shimming algorithms at 3 T will contribute to further improvements of imaging quality and shorter scanning times.
